# Reduced Interleukin-17-Expressing Cells in Cutaneous Melanoma

**DOI:** 10.3390/biomedicines9121930

**Published:** 2021-12-16

**Authors:** Anna Tosi, Lavinia Nardinocchi, Maria Luigia Carbone, Lorena Capriotti, Elena Pagani, Simona Mastroeni, Cristina Fortes, Fernanda Scopelliti, Caterina Cattani, Francesca Passarelli, Antonio Rosato, Stefania D’Atri, Cristina Maria Failla, Andrea Cavani

**Affiliations:** 1 Department of Surgery, Oncology and Gastroenterology, University of Padova, 35128 Padova, Italy; tosi-anna@libero.it; 2 Experimental Immunology Laboratory, Istituto Dermatopatico dell’Immacolata IDI-IRCCS, 00167 Rome, Italy; lavix@inwind.it (L.N.); marialuigia.carbone@idi.it (M.L.C.); capriottilorena@gmail.com (L.C.); c.failla@idi.it (C.M.F.); 3 Molecular Oncology Laboratory, Istituto Dermatopatico dell’Immacolata IDI-IRCCS, 00167 Rome, Italy; elenapagani15@gmail.com (E.P.); s.datri@idi.it (S.D.); 4 Epidemiology Unit, Istituto Dermatopatico dell’Immacolata IDI-IRCCS, 00167 Rome, Italy; s.mastroeni@idi.it (S.M.); c.fortes@idi.it (C.F.); 5 Istituto Nazionale per la Promozione della Salute delle Popolazioni Migranti ed il Contrasto delle Malattie della Povertà INMP, 00153 Rome, Italy; Fernanda.Scopelliti@inmp.it (F.S.); caterina.cattani@inmp.it (C.C.); cavani@inmp.it (A.C.); 6 Histopathology Department, Istituto Dermatopatico dell’Immacolata IDI-IRCCS, 00167 Rome, Italy; f.passarelli@idi.it; 7 Immunology and Molecular Oncology Diagnostics, Veneto Institute of Oncology IOV-IRCCS, 35128 Padova, Italy

**Keywords:** interleukin-17, T-helper 17 lymphocytes, melanoma, tumor infiltrating lymphocytes

## Abstract

Characterization of tumor associated lymphocytes (TILs) in tumor lesions is important to obtain a clear definition of their prognostic value and address novel therapeutic opportunities. In this work, we examined the presence of T helper (Th)17 lymphocytes in cutaneous melanoma. We performed an immunohistochemical analysis of a small cohort of primary melanomas, retrospectively selected. Thereafter, we isolated TILs from seven freshly surgically removed melanomas and from three basal cell carcinomas (BCC), as a comparison with a non-melanoma skin cancer known to retain a high amount of Th17 cells. In both studies, we found that, differently from BCC, melanoma samples showed a lower percentage of Th17 lymphocytes. Additionally, TIL clones could not be induced to differentiate towards the Th17 phenotype in vitro. The presence or absence of Th17 cells did not correlate with any patient characteristics. We only observed a lower amount of Th17 cells in samples from woman donors. We found a tendency towards an association between expression by melanoma cells of placenta growth factor, angiogenic factors able to induce Th17 differentiation, and presence of Th17 lymphocytes. Taken together, our data indicate the necessity of a deeper analysis of Th17 lymphocytes in cutaneous melanoma before correlating them with prognosis or proposing Th17-cell based therapeutic approaches.

## 1. Introduction

The presence of an inflammatory infiltrate of both innate and adaptive immune cells around the tumor has been long recognized and can be interpreted in different ways [1]. It may reflect a protective host response against cancer cells or a persistent tumor-promoted inflammation able to foster fibrosis, angiogenesis, and tumor progression [2].

In cutaneous melanoma, tumor-infiltrating lymphocytes (TILs) can be present focally or diffusively at the tumor periphery and histological samples can be categorized based on TIL density and distribution pattern [3,4]. In the majority of epidemiological studies, the presence of TILs in the primary lesion has been considered a favorable prognostic element in melanoma patients [5,6,7]. A study inversely associated TIL occurrence with the development of sentinel lymph node metastasis [8]. The fact that not all the studies succeeded in demonstrating an association between TIL presence and melanoma favorable outcome could be explained by different events. Chemotherapy and target therapy can affect the local inflammatory and immune microenvironment [9], altering and changing TIL density and distribution during the disease. Moreover, tumor growth is frequently accompanied by local immunosuppression, with the expansion of regulatory T lymphocytes, recruitment of myeloid-derived suppressor cells or tumor associated macrophages, and, finally, induction of functional anergia of the peri-tumoral T lymphocytes [1,10]. Biological mechanisms responsible for immunosuppression involve both secretions of anti-inflammatory cytokines and the expression of immunomodulating membrane molecules on melanoma cells, such as checkpoint receptors [10,11]. Therefore, the occurrence of high amounts of TILs cannot be considered per se an indicator of an active and effective immunological response of the patient against melanoma cells.

The difference in the outcome could be due to the diverse immune cell subsets that composed the TILs or, alternatively, to the induction of TIL anergia by the melanoma cells. Considering this concept, a deeper characterization of the T lymphocyte subtypes in the tumor specimens has been carried out, trying to associate the presence of a specific subtype with the immunological response against melanoma, a greater overall survival, or a better outcome of therapeutic approaches [12,13,14]. In fact, usage of anti-checkpoint inhibitor immunotherapy in metastatic melanoma has underlined the importance of TIL characterization [15] and the need to investigate the relationship between melanoma and immune cells in the tumor microenvironment [11].

TILs include different lymphocyte subsets: CD8+ and CD4+ T cells, natural killer (NK) cells, and B cells. CD8+ cytotoxic lymphocytes inhibit tumor proliferation and a large amount of CD8+ TILs, alone or together with CD4+ T helper (Th)1 cells, has been associated with a good prognosis in cutaneous melanoma [16,17]. CD4+ T cell types also include Th2, Th17, and T regulatory (Treg) lymphocytes.

The Th17 cell subtype, characterized by secretion of interleukin (IL)-17A, has been extensively studied in cutaneous inflammatory processes, and in autoimmune pathologies. However, the role of Th17 in neoplastic growth is controversial [18]. In fact, having great developmental plasticity and depending on the particular cytokine milieu to which they are exposed, Th17 cells can convert towards a Th1 phenotype [19,20], and thus exert a protective function against tumor development. On the other hand, Th17 cells can or switch towards a regulatory phenotype with suppressive function, supporting tumor cell escape from host immune surveillance and tumor growth [21,22].

In melanoma, both the previously indicated cases have been reported (reviewed in [23]. An increase in Th17 cells has been observed in melanomas compared to normal skin [24] or dysplastic naevi [25]. Moreover, adoptive therapy with Th17 lymphocytes has been reported to be capable of eradicating melanoma cells in mice [26,27]. Therefore, a deeper characterization of Th17 cells in cutaneous melanoma has become fundamental.

To investigate the presence and significance of Th17 lymphocytes in human cutaneous melanoma, we started our analysis from a retrospectively selected small cohort of patients and then moved towards the study of TILs isolated from melanoma samples. We compared our results on TILs with those obtained with the peripheral blood mononuclear cells (PBMC) from the same patient and with TILs isolated from basal cell carcinomas (BCC), a skin tumor type characterized by the presence of a high percentage of Th17 cells [28].

## 2. Materials and Methods

### 2.1. Patients

The first analysis was performed on a small cohort, selected in a retrospective way among patients who had undergone surgical removal of a primary cutaneous melanoma, stage I to III, at IDI-IRCCS between 1998 and 2001. Patients selected were enrolled in a previous study and have signed informed consent for similar subsequent studies approved by the Institute Ethical Committee. The selection was based on the availability of all necessary clinical and survival data and histological samples. No other inclusion criteria were used. Histological slides of the primary tumors were obtained from the IDI-IRCCS Histopathology Unit archives and used for immunohistochemical and immunofluorescent analyses. Subsequently, for TIL isolation, a prospective study was performed and patients undergoing surgical removal of primary cutaneous melanoma, or a cutaneous melanoma metastasis or a BCC at IDI-IRCCS between 2013 and 2015 were enrolled. All patients signed informed consent. From the freshly surgically removed specimens, the pathologist selected a 3 mm tissue fragment from which TILs were isolated and characterized. This study was approved by the IDI-IRCCS Ethical Committee (n. 38, 2013) and performed in accordance with the Declaration of Helsinki.

### 2.2. Cell Culture

Six human melanoma cell lines of primary and metastatic origin were used. Two were established in our laboratory (GR-Mel and SN-Mel), two were purchased from the American Type Culture Collection (ATCC, Rockville, MD, USA) (WM115 and WM266-4), and two were a generous gift from Dr. F. Guadagni (Inter Institutional Multidisciplinary Biobank, IRCCS San Raffaele Pisana, Rome, Italy) (LCP-Mel and LCM-Mel). Among these melanoma cell lines, three were from primary tumors (GR-Mel, WM-115, LCP-Mel), two from cutaneous metastases (SN-Mel, WM266-4), and one from a non-cutaneous metastasis (LCM-Mel). Melanoma cells were grown as previously described [29].

### 2.3. Immunohistochemistry

Paraformaldehyde-fixed, paraffin-embedded, 4-μ-thick melanoma sections were deparaffinized, rehydrated, and processed for immunohistochemical analysis. The following primary antibodies were used for staining: anti-human CD3 rabbit polyclonal antibody (A0452, Dako, Glostrup, Denmark), at a concentration of 6 μg/mL; anti-human IL-17A goat polyclonal antibody (AF-317-NA, R&D Systems, Minneapolis, MN, USA), at 1:20 dilution; anti-human placenta growth factor (PlGF) rabbit polyclonal antibody (clone 1880, Santa Cruz Biotechnology, Santa Cruz, CA, USA), at 1:50 dilution. Immunoreactivity was visualized with a peroxidase reaction using 3-amino-9-ethylcarbazole (AEC, Vector Laboratories, Burlingame, CA, USA), and the specimens were counterstained with hematoxylin. Negative controls were obtained by omitting the primary antibody. Stained sections were analyzed with an AxioCam digital camera attached to an Axioplan 2 microscope (Carl Zeiss AG, Oberkochen, Germany). Positive cells were counted in 10 fields per specimen at magnification 200×.

### 2.4. TIL Isolation and Growth

Fresh tumor tissues were washed with RPMI 1640 (Lonza, Basel, Switzerland), and fatty, connective parts were removed. Tissues were minced into little pieces with a sterile scalpel and placed in culture in RPMI 1640 with the addition of 5% human serum and 60 U/mL recombinant human IL-2 (Novartis, Varese, Italy). After 4–7 days, cells emigrated from tissue were collected and placed in starvation with minimal IL-2 supply before phenotypic characterization, functional assays, and T cell cloning by limiting dilution (0.6 cells/well) in the presence of irradiated allogeneic feeder cells plus 1% phytohemagglutinin-M (Roche, Mannheim, Germany). For selected experiments, TIL clones, maintained in culture in RPMI 1640 plus IL-2 (80 U/mL), were stimulated for 5 days with IL-6 (10 µg/µL) and IL-1β (10 µg/µL) (R&D Systems, Minneapolis, MN, USA), following previous indications [30,31,32], and then examined by flow cytometry. PBMC were isolated from whole blood samples of the same patients from which the tumor biopsy was taken by Ficoll-Paque PLUS (Lonza, Basel, Switzerland) centrifugation.

### 2.5. Flow Cytometric Analysis

For intracellular evaluation of cytokine production, TIL clones or PBMC were incubated with phorbol 12-myristate 13-acetate (PMA, 10 ng/mL), ionomycine (1 mg/mL), and monensin (2 mM) (BD Bioscience, East Rutherford, NJ, USA) for 2 h at 37 °C. Thereafter, brefeldin (5 mg/mL) was added to the cells and the incubation was carried out for an additional 4 h. Cells were collected, fixed, and permeabilized with Cytofix/Cytoperm (BD Biosciences, East Rutherford, NJ, USA) and stained for 20 min with the antibodies in the presence of Perm/Wash solution (BD Biosciences, East Rutherford, NJ, USA). Melanoma cells were detached from the plate using a PBS/EDTA solution, washed with 1× PBS, and resuspended into 2 mL 1× PBS. Cells were fixed and stained as described above. In selected experiments, melanoma cells were treated or not for 24 h with TNF-α (50 ng/mL) and IFN-γ (200 U/mL) (R&D Systems, Minneapolis, MN, USA) and then examined. Acquisition and analysis were done using a FACSAria equipped with Diva software (version 6.0; BD Biosciences, East Rutherford, NJ, USA). Monoclonal antibodies used for intra cytoplasmic cytokines or membrane antigen detection were as follows: Tumor Necrosis Factor (TNF)-α-FITC, interferon (IFN)-γ-PB, CD4-FITC, CD8-PB, CD3-allophycocyanin-Cy7 (BD Biosciences, East Rutherford, NJ, USA); IL-17A-PE and IL-17RA-PE (EBiosciences, Frankfurt, Germany); IL-22-allophycocyanin and IL-22RA1-allophycocyanin (R&D Systems, Minneapolis, MN, USA).

### 2.6. ELISA

TIL clones were cultured in RPMI 1640 plus IL-2 (80 U/mL) (Lonza, Basel, Switzerland), and stimulated for 48 h with anti-CD3 and anti-CD28 antibodies. Cell culture supernatants were collected and measured for their content of TNF-α, IFN-γ, IL-4, IL-8, IL-17A, IL-22, and PlGF using the duoset kit of R&D Systems (Minneapolis, MN, USA) following the manufacturer’s protocol.

### 2.7. Real-Time RT-PCR

Total RNA was extracted from TILs by TRIZOL reagent (Invitrogen-Life Technologies, Carlsbad, CA, USA), and further purified using RNeasy mini kit (Qiagen, Germantown, MD, USA) and DNAse I treatment (Qiagen, Germantown, MD, USA). cDNA synthesis was performed using the SuperScript III First Strand System (Invitrogen-Life Technologies, Carlsbad, CA, USA) accordingly to the manufacturer’s protocol. Real-time PCR was performed using the TaqMan assays-gene expression (ThermoFisher, Waltham, MA, USA) for IL-22 (Hs01574152_g1), IL-8 (Hs00174103_m1), GATA3 (Hs00231122_m1), RORC (Hs01076112_m1), and 18S (Hs03003631_g1) as a normalization control. Real-time RT-PCR was performed in triplicate using an ABI 7000 thermal cycler (PerkinElmer, Groningen, The Netherlands). Quantitative calculations were performed using the formula 2^−ΔCt^. To simplify data presentation, the relative expression values were multiplied by 10^5^.

### 2.8. Multiplex Fluorescence Immunohistochemistry

This analysis was carried out on 4 µm-thick formalin-fixed paraffin-embedded tissue sections using the Opal 7-colors manual IHC kit (Akoya Biosciences, Marlborough, MA, USA). Slides were deparaffinized and rehydrated by serial passages in clearance solvent (Leica Biosystems, Milton Keynes, United Kingdom) and graded ethanol. Two staining panels were used to characterize the subsets of tumor infiltrating immune cells. The first panel included antibodies directed against human IL-17A (polyclonal, Abcam, Cambridge, UK), CD56 (clone 123C3, Agilent, Santa Clara, CA, USA), neutrophil elastase (clone NP57, Agilent, Santa Clara, CA, USA), CD4 (clone 4B12, ThermoFisher, Waltham, MA, USA), CD3 (clone F.7.2.38, Agilent, Santa Clara, CA, USA), and a melanoma mix consisting of the anti-melanoma antibody cocktail (clone HMB45 + M2-7C10 + M2-9E3 + T311, Abcam, Cambridge, UK) and Sox-10 (EP268, Epitomics, Darmstadt, Germany). The second panel comprised antibodies directed against human PlGF (clone 1880, Santa Cruz Biotechnology), IL-17A (polyclonal, Abcam, Cambridge, UK), FoxP3 (clone D2W8E, Cell Signaling Technology, Danvers, MA, USA), CD11b (clone EP1345Y, Abcam, Cambridge, UK), CD8 (clone C8/144B, Agilent, Santa Clara, CA, USA) and the melanoma mix used in the first panel. Heat-induced epitope retrieval was performed in a microwave oven using Target Retrieval Solution pH9 (Agilent, Santa Clara, CA, USA) or pH6 (Akoya Biosciences, Marlborough, MA, USA), depending on the primary antibody. The staining procedure consisted of sequential rounds of protein blocking with Protein Block Serum-free (Agilent), followed by primary antibody and secondary horseradish peroxidase (HRP)-conjugated antibody that mediates the covalent binding of a different Tyramide Signal Amplification (TSA)-conjugated Opal fluorophore (Akoya Biosciences, Marlborough, MA, USA) to the antigen. Finally, slides were counterstained with DAPI supplied by the kit, and mounted with Vectashield fluorescence mounting medium (Vector Labs, Burlingame, CA, USA). Multiplex-stained slides were scanned at 20× using the Mantra Quantitative Pathology Workstation (Akoya Biosciences, Marlborough, MA, USA) and analyzed with InForm Image Analysis Software (Akoya Biosciences, v2.4.10). For each sample, only areas comprising tumor cells were considered, to avoid the acquisition and analysis of normal tissues. Cell density was reported as the mean of all acquired fields from the same tissue slide (at least 20 fields at magnification 20× for each stained slide).

### 2.9. Statistical Analysis

Data were described in terms of sex, age, histological and clinical characteristics, number of IL-17A-positive cells, the intensity of PlGF staining in the tumor, vessels, and inflammatory infiltrate. Features were presented as median and Interquartile Range (IQR) for continuous variables (i.e., age and Breslow thickness) and as absolute numbers and percentages for categorical variables. Age in years was presented as a continuous variable and as a categorical variable (<60 and ≥60); Breslow thickness as a continuous variable and as a categorical variable (≤4.00 and ≥4.01 mm). The anatomic site of localization was categorized as “head/neck”, “trunk”, “limbs”; histological type as “superficial spreading” and “nodular”; mitotic rate as ≤1 mitosis per mm^2^ (low) and ≥1 mitoses per mm^2^ (high); the presence of ulceration as “no”/“yes”; cell type as “epithelioid” and “other”; TILs as “scantly”, “moderate” and marked [33], and, when available, sentinel lymph node status as “negative”/“positive”. The number of IL-17A-positive cells was categorized in tertiles based on the distribution as follows: ≤10 (low), 10.1–17.9 (medium), ≥18 (high). PlGF staining as “no”/“yes”. Associations between characteristics, IL-17A or PlGF expression were assessed using the Kruskal–Wallis test and the Fisher’s exact test for continuous and categorical variables, respectively. The Kaplan–Meier method was used to estimate melanoma specific survival. All analyses were performed using Stata Statistical Software: Release 15. College Station, TX, USA: StataCorp LLC. Statistical analysis of flow cytometry data was performed using Student’s *t*-test by GraphPad prism Software (version 8.4.3; La Jolla, CA, USA).

## 3. Results

### 3.1. Low Amounts of IL-17A-Expressing Cells Are Present among the TILs

Twenty-six patients with cutaneous melanoma who had undergone surgical excision of their primary lesion at IDI-IRCCS between 1998 and 2001 were analyzed for the retrospective study. These patients were selected as they were included in a previous similar study and they had signed an informed consensus. All the patients examined were treatment naïve. In the study population, there were 13 deaths, eight of which were due to melanoma. The median follow-up time was 8.1 years for a total of 163.3 person-year of follow-up. Ten-year melanoma specific survival was 66.8%.

Immunohistochemical analysis was performed using an anti-CD3 antibody, to identify T lymphocytes, and an anti-IL-17A, to count the IL-17A-expressing cells. The presence of a slight or moderate T lymphocyte infiltrate was observed in 16 and 10 patients, respectively. Moreover, modest staining with the anti-IL-17A antibody was seen inside and at the boundary of the tumor lesions (Supplementary Figure S1). As shown in Table 1, no association was found between demographic, histological, or clinical characteristics of the patients and IL-17A expression, except for sex. Females showed a lower expression of IL-17A (*p*-value: 0.026) in comparison with males.

TILs were then isolated from fragments of melanoma specimen surgically removed from seven different patients, either primary lesions (two cases) or metastatic samples (five cases). Additionally, in this case, patients were treatment naïve at the time of surgical removal of the lesion. Table 2 reports the characteristics of the patients from whose tumor TILs were extracted. TILs were cultured in the presence of IL-2, cloned to limited dilution, and analyzed by flow cytometry. The relative percentage of the different subtypes of T lymphocytes was compared with peripheral blood mononuclear cell (PBMC) subtypes of the same individual. As a comparison with a different skin tumor type known to have a large amount of Th17 cells among TILs, the data were also compared to TILs isolated from three different BCC biopsies.

**Figure 1:**
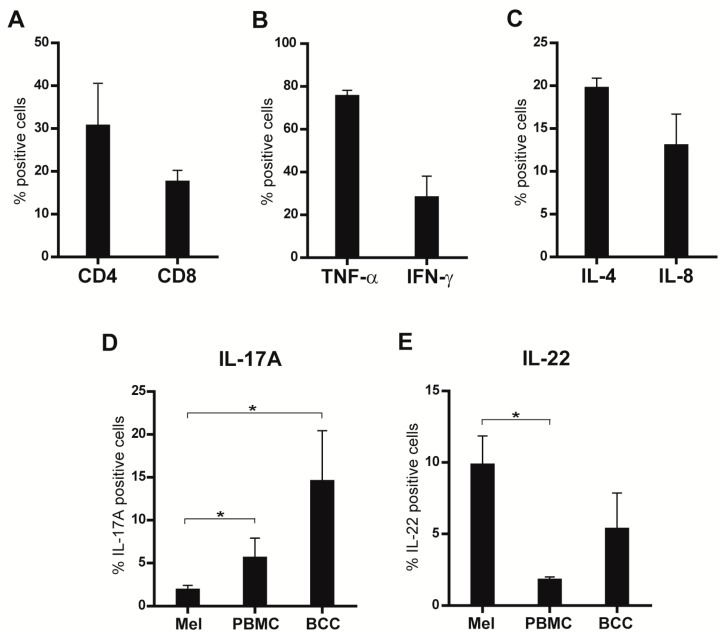
TIL clones isolated from melanoma biopsies were analyzed for (**A**) CD4 and CD8; (**B**) TNF-α, IFN-γ; (**C**) IL-4 and IL-8; (**D**) IL-17A; (**E**) IL-22 expression by flow cytometry. Results are shown as the mean of the percentage ± standard error of the mean (SEM) of the data from the seven patients. In (**D**,**E**), the percentage of positive TILs from the melanoma biopsies was compared with that of the PBMCs from the same patient or with that of TILs isolated from BCCs. * *p* < 0.05; Student’s *t*-test.

The median frequency of the TIL clones extracted from the seven patients showed enrichment in CD4+ T lymphocytes (30.6% ± 16) with a lower number of CD8+ cells (17.5% ± 4.6) (Figure 1A). TIL clones expressed tumor necrosis factor (TNF)-α (74% ± 6), interferon (IFN)-γ (28% ± 23), IL-4 (20% ± 2.5), IL-8 (13% ± 9), IL-17A (1.9% ± 1.3) ed IL-22 (9.7% ± 4.8), at a variable grade (Figure 1B–E). The median number of IL-17A+ cells isolated from the tumor tissue was significantly lower with respect to IL-17A-expressing cells in the PBMC (5.6% ± 3.2) and to IL-17A+ TILs isolated from BCC biopsies (19.5% ± 7) (Figure 1D). On the other hand, the number of IL-22+ TILs from the melanoma samples was higher than that of the PBMC (1.8% ± 0.3) and analogous to the number of IL-22+ TILs isolated from BCC samples (9.6% ± 1.3) (Figure 1E).

**Figure 2:**
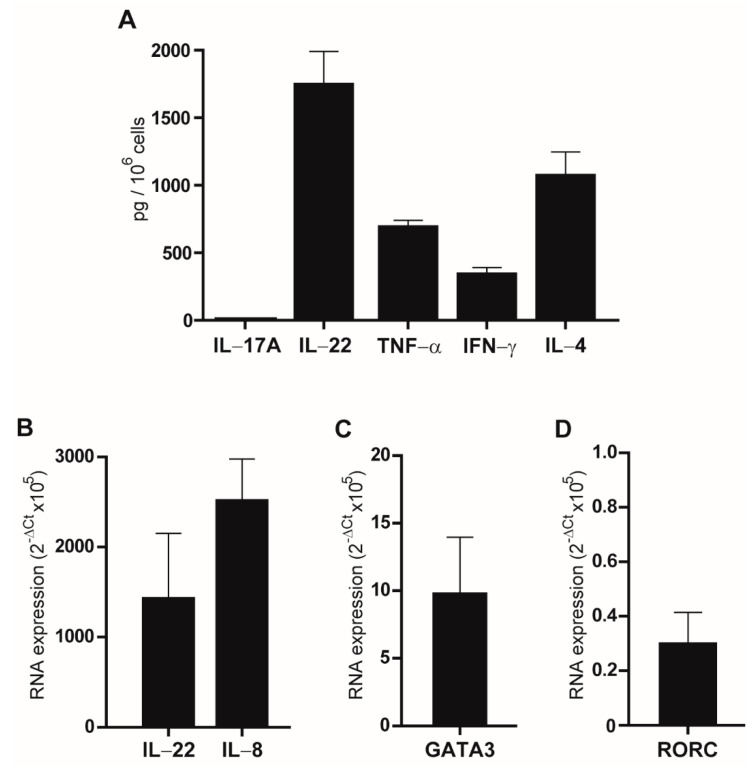
Analyses of the TIL clones. (**A**) ELISA was performed on the supernatant of the clones following stimulation with anti-CD3 and anti-CD28 antibodies for 48 h. The indicated cytokines were analyzed. Results are reported as mean ± standard error of mean (SEM) of duplicate samples from three experiments. (**B**–**D**) Real-time RT-PCR of the clones. Basal expression of IL-22, IL-8, GATA3, and RORC is reported as mean of 2^−ΔCt^ × 10^5^ values of three independent experiments ± SEM.

Limited dilution gave up to 50 T-cell clones, all CD4+, with low efficiency (0.02%), probably reflecting a slight expanding attitude of peri-tumor T lymphocytes in in-vitro culture conditions. ELISA was performed on the supernatant of the 50 clones stimulated for 48 h with anti-CD3 and anti-CD28 antibodies. Secretion of IL-17A, IL-22, TNF-α, IFN-γ and IL-4, was evaluated and confirmed that IL-17A was detectable only in two T cell clones. Secretion of TNF-α was always detected whereas three, seven and seven clones out of 50 did not secrete IFN-γ, IL-4, and IL-22, respectively. Figure 2A shows the mean values of the 50 clones.

To evaluate whether TILs could be induced in vitro to produce IL-17A, three different clones were cultured and stimulated for 5 days with IL-6 and IL-1β, two cytokines involved in Th17 polarization [30]. However, subsequent FACS analyses did not show any differences in the percentage of IL-17A-expressing cells in stimulated versus non-stimulated lymphocytes (data not shown).

The mRNA was isolated from these three clones and analyzed by real-time RT-PCR to evaluate the expression of IL-17A, IL-8, IL-22, GATA3, a transcription factor highly involved in the regulation of IL-4 and consequently a modulator of Th17 responses [34], and RORC, a transcription factor involved in IL-17A production by Th-17 cells [35]. As reported in Figure 2B–D, GATA3, as well as IL-8 and IL-22 expression, was highly variable in the examined clones, whereas IL-17A transcript was undetectable (data not shown). Low levels of RORC expression were observed.

Altogether, these data indicated that low Th-17 cells were present in human melanoma samples in respect to other skin tumor types.

### 3.2. Low Amount of Th-17 Cells Are Present in the Original Melanoma Biopsies

**Figure 3:**
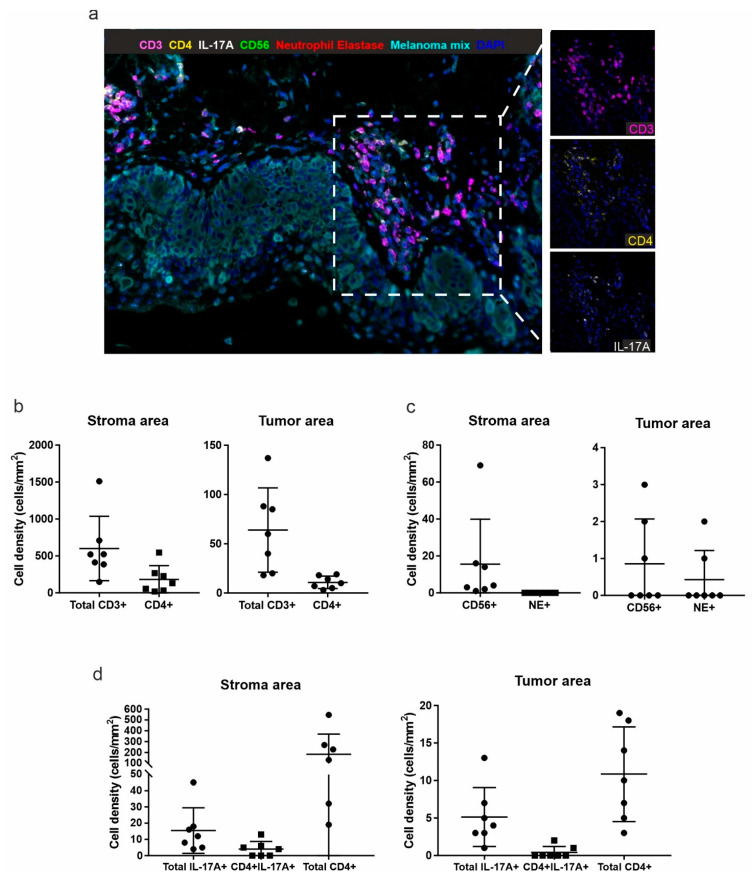
Multiplex immunofluorescence analysis of the first panel. (**a**) Representative 7-color multispectral image of the first multiplex immunofluorescence panel. Markers and color codes are indicated above the figure. Original magnification 20×. Right panels: crops showing only CD3+ (magenta), CD4+ (yellow) and IL-17A+ (white) cells. Density (number of cells/mm^2^) of total CD3+ and CD3+CD4+ cells (**b**), CD56+ cells and Neutrophil elastase+ (NE+) cells (**c**), total IL-17A+ cells, CD4+IL-17A+ and total CD4+ cells (**d**) was quantified in the stroma (left panels) and in the intra-tumoral (right panels) regions. Each dot represents the mean of all acquired fields from the same tissue sample (at least 20 fields at magnification 20× for each stained slide).

To verify that the characterization of TILs isolated from the melanoma biopsies was representative of the real in vivo situation, multiplex fluorescence immunohistochemistry was performed on the histological samples from which the clones were derived. Overall, melanoma samples disclosed a detectable albeit variable immune infiltrate (Figure 3a).

The quantification of immune cell populations was performed considering only the stromal or the intra-tumoral regions in each patient sample. Most immune cells were found in the stroma area. Among the total CD3+ T lymphocytes, the proportion of CD4+ T cells was about 36% in the stroma, and less than 20% within the tumor mass (Figure 3b). However, due to the technical difficulty to detect CD4+ T cells by immunohistochemistry, these percentages could be underestimated. Very few neutrophils (neutrophil elastase+ cells, NE+) and natural killer cells (CD56+) were detected in both regions (Figure 3c). Moreover, we found cells co-expressing IL-17A and CD4 both in the stroma and tumor regions, which accounted for 22% and 17% of all IL-17A+ cells, respectively, and for 2% and 6% of all CD4+ cells, respectively (Figure 3d). Conversely, IL-17A expression was not found in both CD56+ natural killer cells and NE+ neutrophils (data not shown). No difference was observed for IL-17A expression between TILs derived from primary and metastatic lesions.

These data supported the previous indication that a low number of Th-17 cells were present in melanoma samples and that this aspect was not due to TIL extraction methods.

### 3.3. Placenta Growth Factor Expression in Melanoma Does Not Support Differentiation of IL-17A-Expressing Cells

Recently, the pro-angiogenic placenta growth factor (PlGF) was reported to be one of the factors able to regulate the differentiation of Th17 lymphocytes [36]. We previously showed that PlGF is expressed by melanoma cells and that this expression increased with disease progression [29]. Therefore, we decided to investigate whether an association could exist in the examined samples between PlGF expression by melanoma cells and the presence of Th17 cells.

**Figure 4:**
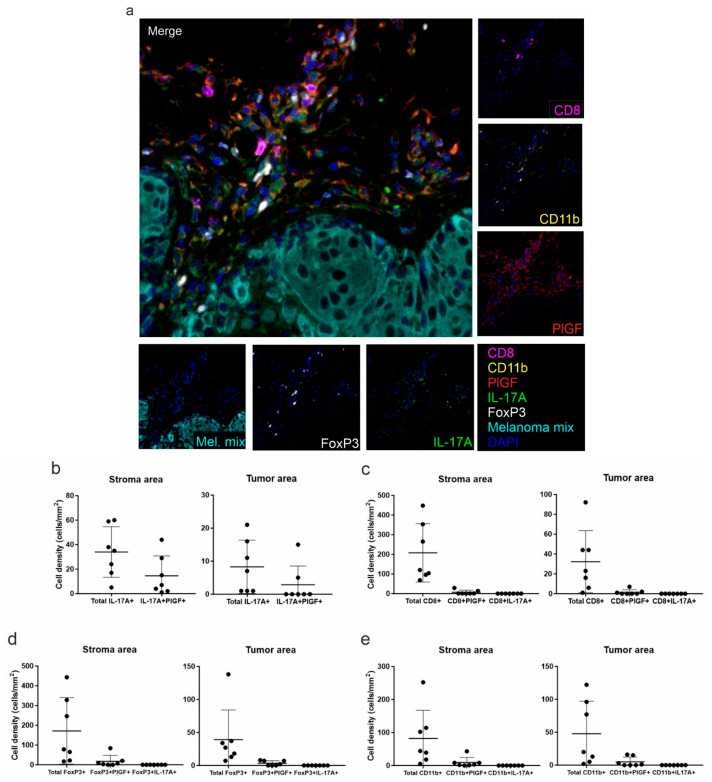
Multiplex immunofluorescence analysis of the second panel. (**a**) Representative 7-color multispectral image of the second multiplex immunofluorescence panel. Markers and color codes are indicated in the figure. Original magnification 20×. Single marker assessment is reported in the small pictures around the merged image. Density (number of cells/mm^2^) of total IL-17A+ and IL-17A+PlGF+ cells (**b**), total CD8+ T lymphocytes, CD8+PlGF+ and CD8+IL-17A+ cells (**c**), total FoxP3+ T regulatory cells, FoxP3+PlGF+ and FoxP3+IL-17A+ cells (**d**), and total CD11b+, CD11b+PlGF+ and CD11b+IL-17A+ cells (**e**) was quantified in the stroma (left panels) and in the intra-tumoral (right panels) regions. Each dot represents the mean of all acquired fields from the same tissue sample (at least 20 fields at magnification 20× for each stained slide).

A second multiplex immunofluorescence panel was designed to assess PlGF expression in the melanoma samples from which the TILs were derived. In all tissue sections, PlGF+ cells were detected at variable levels and prevalently in the stroma (Figure 4a). The nature of PlGF+ cells was also investigated. Among total IL-17A+ cells, 43% and 17% were double positive for PlGF in the stroma and within the tumor masses, respectively (Figure 4b). Considering the lymphoid populations, PlGF expression was also found in a small proportion of CD8+ T lymphocytes (Figure 4c), and in the 12% and 16% of stromal and intra-tumoral FoxP3+ T regulatory cells, respectively (Figure 4d). Similar results were observed considering the CD11b+ population (Figure 4e).

**Figure 5:**
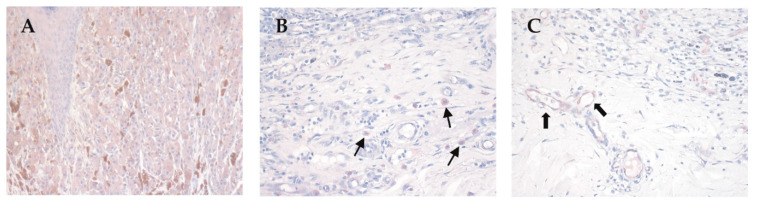
Immunohistochemical analysis for PlGF expression in melanoma samples. Tumor cells were positive for PlGF (**A**), as well as inflammatory infiltrate (**B**) and vessels surrounding the tumor mass (**C**). Thin arrows in B indicate PlGF+ inflammatory in Figure 200×.

The primary melanomas from the 26 patients of the retrospective study were further evaluated by immunohistochemistry for PlGF expression. As shown in Figure 5, positive cytoplasmic staining was observed in melanoma cells (Figure 5A), in cells of the inflammatory infiltrate at the tumor boundaries (Figure 5B), and in dermal vessel endothelial cells (Figure 5C).

As shown in Table 3, no association between patient clinical characteristics and PlGF expression was observed. No association was found between PlGF-expressing vessels or PlGF-expressing inflammatory infiltrate and IL-17A-expressing cells (Fisher’s exact test, *p* = 0.179 and *p* = 0.885, respectively). High expression levels of PlGF in tumor cells were more frequently associated with a high level of IL-17A-expressing inflammatory cells, but the association did not reach statistical significance (*p* = 0.067).

These analyses indicated that PlGF expression in melanoma cells or in the tumor microenvironment did not directly favor Th-17 cell differentiation or recruitment.

### 3.4. Melanoma Cell Lines Did Not Express IL-17A Receptor In Vitro

Previously published data indicated that murine and human melanoma cell lines expressed the IL-17 receptor (R) and responded to IL-17A stimulation [30]. As we have previously shown that different skin tumor types proliferated in response to IL-17A treatment both in vitro and in vivo [28], we evaluated by flow cytometry expression of IL-17 receptor (R)A in six human melanoma cell lines. Of the six melanoma cell lines examined, none expressed IL-17RA in basal conditions (Supplementary Figure S2). To verify whether melanoma cells could be induced to express IL-17RA, cells were stimulated with the inflammatory TNF-α and IFN-γ cytokines for 24 h and flow cytometric analysis was repeated. No difference in receptor expression was observed (not shown). As a control, flow cytometry was also performed on normal human primary keratinocytes and fibroblasts. IL-17RA was expressed by both cell types (Supplementary Figure S2).

Therefore, in our experimental conditions, human melanoma cells did not express IL-17RA in vitro.

## 4. Discussion

Immune responses against a tumor are mounted by different immune cells, but T lymphocytes remain the main mediators of anti-cancer immunity, and a strong CD8+ effector T cell response is necessary to obtain tumor eradication. Either CD4+ Th1 or Th17 cells promote CD8+ cells activation [27], and a lower infiltration of IL-17A-expressing cells could indicate a diminished anti-tumor response. Our analyses showed a reduced presence of IL-17A-expressing TILs in cutaneous melanoma with respect to other skin tumors, such as squamous cell carcinoma [28] or BCC. This result was unexpected, as Th17 lymphocytes were previously detected in other human tumor tissues, such as ovarian, pancreatic, and renal cell carcinoma, as well as in a mouse model of melanoma [37] and in some human melanoma cell lines [24].

Dynamic differentiation of IL-17A-expressing T cells in the tumor, due to the presence of particular mediators, such as IL-2, RANTES, or MCP-1, has been demonstrated [21,24,37]. Therefore, there is the possibility that human melanoma expresses a combination of factors able to restrict T cell differentiation towards the Th17 phenotype or inhibit Th17 recruitment at the tumor site. In ovarian cancer, the levels of Th17 TILs positively correlate with patient outcome [38], and therefore, the inhibition of Th17 lymphocytes may represent a novel mechanism for the tumor to escape immune surveillance. It remains to be clarified which molecular processes address melanoma cells towards this kind of immune suppression and if it is dependent on particular patient characteristics.

An additional hypothesis for such specific immunosuppression performed by melanoma cells in respect to Th-17 lymphocytes could be that melanoma cells were inhibited by IL-17A, and therefore, have found a mechanism to block IL-17A-producing cells. However, in our hands, melanoma cells did not express IL-17RA in vitro and would not respond to IL-17A stimulation.

Differently from what we observed in cutaneous melanoma samples, Th17 lymphocytes are particularly abundant in the skin in inflammatory pathologies, such as psoriasis and eczema, and in skin tumors of keratinocyte origin [28]. Interestingly, patients affected by psoriasis, where Th17 cells have a clear pathogenic role, exhibit a lower incidence of melanoma [39]. The presence in the skin of Th17 lymphocytes could be involved in the protection of normal melanocytes from neoplastic transformation or could reduce melanoma cell growth. However, since psoriasis patients show a lower incidence of non-melanoma skin cancer as well [40], despite the higher IL-17A presence in such a tumor microenvironment, probably other psoriasis-related factors are involved in sustaining the positive immune surveillance against skin tumor development, and Th-17/IL-17A presence could be a consequence instead of the leading cause of this immune surveillance.

In our study, IL-17A expression in melanoma samples was not associated with any histological or clinical parameter. However, our patient cohort is too small to exclude any significant association between IL-17A expression and prognosis. We only noted that females presented lower expression of IL-17A in comparison to males. Interestingly, the correlation of TIL presence in tumor samples and prognostic features, such as sentinel lymph node metastases, was previously reported to be differently relevant between men and women [33,41]. Nevertheless, no data have been presented for a sex-dependent relationship between Th17 lymphocytes and prognosis.

In the histological specimens examined, we did not detect a direct IL-17A expression by melanoma cells as was previously reported [42]. However, we observed that, in addition to Th17, other inflammatory cells express IL-17A in the tumor tissue. These cells do not belong to the FoxP3+ T-reg or the CD11b+ cell or the neutrophil subsets and their identity should be better investigated in the future.

In an attempt to find possible factors related to Th17 differentiation within the tumor tissue, we analyzed PlGF secretion in melanoma cells and in the tumor microenvironment of our samples, since this angiogenic factor is expressed in melanoma [29] and was involved in the generation of Th-17 cells [36]. As previously reported [36], we found that a number of infiltrating leukocytes express both PlGF and IL-17A, but the association between PlGF secretion and IL-17A-expressing cells did not reach statistical significance. Therefore, to better clarify if PlGF could have a role in the differentiation of Th-17 cells in melanoma, a larger number of patients should be considered.

The crosstalk between angiogenic growth factors of the vascular endothelial growth factor family, such as PlGF, and IL-17A-expressing cells, has been underlined by us and other authors [36,43,44]. This aspect, together with the possible mechanisms induced by melanoma cells to block Th-17 cell differentiation or recruitment, should be deeper examined and clarified before considering the application of the promising Th17-based adoptive transfer immunotherapy in the treatment of cutaneous melanoma.

## Figures and Tables

**Table 1:** Demographic, histological, and clinical characteristics of patients and IL-17A levels.

Characteristics	All	IL-17A Low (≤10.0)	IL-17A Medium (10.1–17.9)	IL-17A High (≥18.0)	*p* Value ^b^
	N = 26	(n = 9)	(n = 9)	(n = 8)	
	N ^a^ (%)	N ^a^ (%)	N ^a^ (%)	N ^a^ (%)	
Sex					
Males	12 (46.1)	1 (11.1)	5 (55.6)	6 (75.0)	
Females	14 (53.9)	8 (88.9)	4 (44.4)	2 (25.0)	0.026
Age, years					
median (IQR)	61 (49–73)	55 (46–81)	60 (41–68)	65 (54–72)	0.576 ^c^
<60	12 (46.1)	5 (55.6)	4 (44.4)	3 (37.5)	
≥60	14 (53.9)	4 (44.4)	5 (55.6)	5 (62.5)	0.885
Breslow thickness, mm					
median (IQR)	5.0 (3.3–8.0)	7.0 (5.0–9.0)	5.0 (4.0–5.0)	4.5 (2.9–6.3)	0.402 ^c^
≤4.00	9 (34.6)	2 (22.2)	3 (33.3)	4 (50.0)	
≥4.01	17 (65.4)	7 (77.8)	6 (66.7)	4 (50.0)	0.521
Anatomic site					
Head/neck	3 (11.5)	2 (22.2)	1 (11.1)	0 (-)	
Trunk	10 (38.5)	3 (33.3)	4 (44.4)	3 (37.5)	
Limbs	13 (50.0)	4 (44.4)	4 (44.4)	5 (62.5)	0.894
Histological type					
superficial spreading	9 (36.0)	2 (22.2)	5 (62.5)	2 (25.0)	
nodular	16 (64.0)	7 (77.8)	3 (37.5)	6 (75.0)	0.264
Mitotic rate					
low (<1 mitosis/mm^2^)	4 (25.0)	2 (28.6)	1 (20.0)	1 (25.0)	
high (≥1 mitoses/mm^2^)	12 (75.0)	5 (71.4)	4 (80.0)	3 (75.0)	1
Presence of ulceration					
no	17 (65.4)	6 (66.7)	4 (44.4)	7 (87.5)	
yes	9 (34.6)	3 (33.3)	5 (55.6)	1 (12.5)	0.264
Cell type					
epithelioid	20 (80.0)	8 (88.9)	6 (75.0)	6 (75.0)	
other	5 (20.0)	1 (11.1)	2 (25.0)	2 (25.0)	0.696
TILs					
scantly	10 (76.9)	4 (100.0)	2 (50.0)	4 (80.0)	
moderate	3 (23.1)	0 (-)	2 (50.0)	1 (20.0)	
marked	0 (-)	0 (-)	0 (-)	0 (-)	0.441
Sentinel lymph node status					
negative	7 (70.0)	2 (50.0)	2 (66.7)	3 (100.0)	
positive	3 (30.0)	2 (50.0)	1 (33.3)	0 (-)	0.700

*IQR*, Interquartile Range; ^a^ totals may vary because of missing value; ^b^ Fisher’s exact test; ^c^ Kruskal–Wallis test.

**Table 2:** Demographic, histological and clinical characteristics of patients from which TILs have been extracted.

Patient	Sex	Age at Diagnosis	Anatomic Site	Breslow Thickness	Histological Type	Mitotic Rate ^a^	Ulceration	Extraction Biopsy	BRAF Mutation
1	F	49.3	trunk	2.5 mm	SSM	high	no	primary	yes
2	M	55.5	lower limb	1.4 mm	nodular	high	no	metastasis	yes
3	F	84.6	lower limb	5.0 mm	SSM	high	yes	metastasis	yes
4	M	41.4	trunk	1.1 mm	SSM	high	no	primary	no
5	M	64.6	lower limb	4.9 mm	nodular	high	yes	metastasis	no
6	F	39.9	lower limb	2.5 mm	nodular	high	no	metastasis	no
7	M	58.4	trunk	0.5 mm	SSM	NA	no	metastasis	no

F, females; M, males; SSM, superficial spreading melanoma; NA, not available. ^a^ defined as low (<1 mitosis/mm^2^), high (≥1 mitoses/mm^2^).

**Table 3:** Characteristics of patients and PlGF expression.

Characteristics	Tumor PlGF-Expressing Cells			*p* Value ^b^	PlGF-Expressing Vessels		*p* Value ^b^	PlGF-Expressing Infiltrating Cells		*p* Value ^b^
	Low (n = 10)	Medium (n = 10)	High (n = 6)		Yes (n = 22)	No (n = 4)		Yes (n = 11)	No (n = 15)	
	N ^a^ (%)	N ^a^ (%)	N ^a^ (%)		N ^a^ (%)	N ^a^ (%)		N ^a^ (%)	N ^a^ (%)	
Sex										
Males	5 (50.0)	3 (30.0)	4 (66.7)		12 (54.6)	0 (-)		5 (45.5)	9 (60.0)	
Females	5 (50.0)	7 (70.0)	2 (33.3)	0.372	10 (45.5)	4 (100.0)	0.100	6 (54.6)	6 (40.0)	0.692
Age, years										
median (IQR)	49 (41–75)	67 (54–77)	64 (55–69)	0.344 ^c^	61 (49–73)	52 (48–65)	0.570 ^c^	68 (55–75)	54 (46–72)	0.204 ^c^
<60	6 (60.0)	4 (40.0)	2 (33.3)		9 (40.9)	3 (75.0)		4 (36.4)	8 (53.3)	
≥60	4 (40.0)	6 (60.0)	4 (66.7)	0.682	13 (59.1)	1 (25.0)	0.306	7 (63.6)	7 (46.7)	0.453
Breslow thickness, mm										
median (IQR)	5.0 (4.5–8.0)	4.5 (2.5–9.0)	5.0 (4.0–7.0)	0.840 ^c^	5.0 (3.3–7.0)	6.9 (3.9–8.5)	0.617 ^c^	5.0 (2.5–10.0)	5.0 (4.0–7.0)	0.876 ^c^
≤4.00	2 (20.0)	5 (50.0)	2 (33.3)		8 (36.4)	1 (25.0)		5 (45.5)	4 (26.7)	
≥4.01	8 (80.0)	5 (50.0)	4 (66.7)	0.344	14 (63.6)	3 (75.0)	1	6 (54.6)	11 (73.3)	0.419
Anatomic site										
Head/neck	1 (10.0)	2 (20.0)	0 (-)		2 (9.1)	1 (25.0)		0 (-)	3 (20.0)	
Trunk	5 (50.0)	3 (30.0)	2 (33.3)		10 (45.5)	0 (-)		6 (54.6)	4 (26.7)	
Limbs	4 (40.0)	5 (50.0)	4 (66.7)	0.764	10 (45.5)	3 (75.0)	0.196	5 (45.4)	8 (53.3)	0.366
Histological type										
superficial spreading	3 (30.0)	2 (22.2)	4 (66.7)		8 (38.1)	1 (25.0)		5 (45.5)	4 (28.6)	
nodular	7 (70.0)	7 (77.8)	2 (33.3)	0.239	13 (61.9)	3 (75.0)	1	6 (54.6)	10 (71.4)	0.434
Mitotic rate										
low (<1 mitosis/mm^2^)	2 (28.6)	1 (20.0)	1 (25.0)		2 (16.7)	2 (50.0)		0 (-)	4 (40.0)	
high (≥1 mitoses/mm^2^)	5 (71.4)	4 (80.0)	3 (75.0)	1	10 (83.3)	2 (50.0)	0.245	6 (100.0)	6 (60.0)	0.234
Presence of ulceration										
no	6 (60.0)	8 (80.0)	3 (50.0)		15 (68.2)	2 (50.0)		8 (72.7)	9 (60.0)	
yes	4 (40.0)	2 (20.0)	3 (50.0)	0.581	7 (31.8)	2 (50.0)	0.591	3 (27.3)	6 (40.0)	0.683
Cell type										
epithelioid	9 (90.0)	7 (77.8)	4 (66.7)		16 (76.2)	4 (100.0)		8 (72.7)	12 (85.7)	
other	1 (10.0)	2 (22.2)	2 (33.3)	0.581	5 (23.8)	0 (-)	0.549	3 (27.3)	2 (14.3)	0.623
TILs										
scantly	3 (60.0)	4 (80.0)	3 (100.0)		7 (70.0)	3 (100.0)		4 (80.0)	6 (75.0)	
moderate	2 (40.0)	1 (20.0)	0 (-)		3 (30.0)	0 (-)		1 (20.0)	2 (25.0)	
marked	0 (-)	0 (-)	0 (-)	0.738	0 (-)	0 (-)	0.528	0 (-)	0 (-)	1
Sentinel lymph node status										
negative	2 (66.7)	4 (80.0)	1 (50.0)		6 (75.0)	1 (50.0)		5 (100.0)	2 (40.0)	
positive	1 (33.3)	1 (20.0)	1 (50.0)	1	2 (25.0)	1 (50.0)	1	0 (-)	3 (60.0)	0.167

*IQR*, Interquartile Range; ^a^ totals may vary because of missing value; ^b^ Fisher’s exact test; ^c^ Kruskal-Wallis test.

## Data Availability

Data presented in this study are available.

## Supplementary Materials

The following are available online at https://www.mdpi.com/article/10.3390/biomedicines9121930/s1, Figure S1: Expression of IL-17A in melanoma samples. Figure S2: Normal human keratinocytes and fibroblasts express IL-17RA. Materials and Methods.
