# Supplementary material for: Reduced Interleukin-17-Expressing Cells in Cutaneous Melanoma
Source: Biomedicines. 2021 Dec 16;9(12):1930. doi: 10.3390/biomedicines9121930 (PMC8698827; doi:10.3390/biomedicines9121930)
Supplement: Supplementary file 1 [file biomedicines-09-01930-s001.zip › biomedicines-1489136-supplementary.pdf]

Supplementary Figure 1

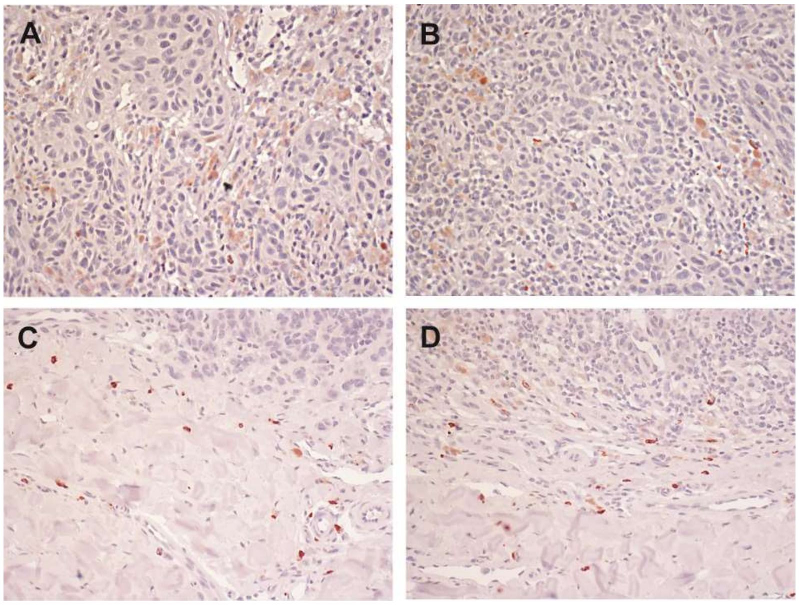

Figure S1. Expression of IL-17A in melanoma samples. IL-17A-positive cells were detected within the tumor (A-B) and at the tumor periphery (C-D). A representative sample is shown. Magnification 200x.

Supplementary Figure 2

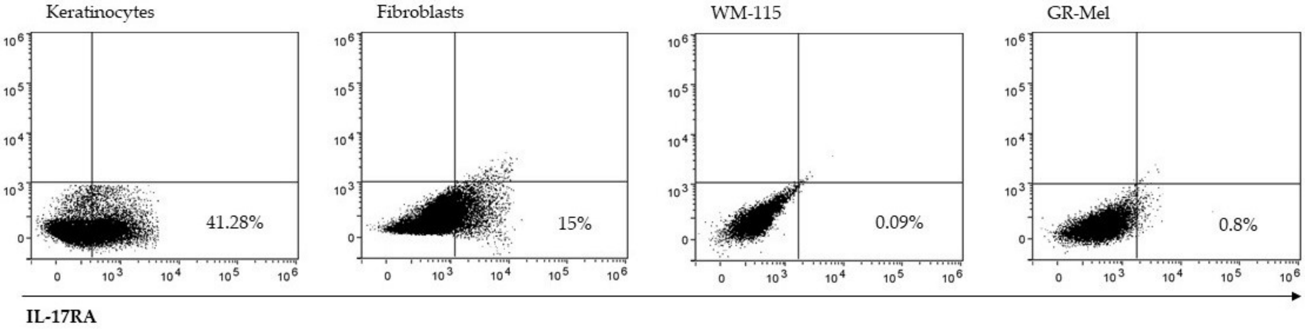

Figure S2. Normal human keratinocytes and fibroblasts express IL-17RA. Flow cytometric analysis of normal human keratinocytes, fibroblasts, and two melanoma cell lines using an anti-IL-17RA antibody. Data from a representative analysis are shown.

Materials and Methods

Primary cultures of human keratinocytes were obtained from skin biopsies, as described previously [1]. Keratinocyte cultures were grown in serum-free keratinocyte growth medium (KGM; Clonetics, Walkersville, MD, USA). Experiments were performed on keratinocyte cultures undergoing terminal differentiation, achieved by growing cells at 100% of confluence. ATCC® normal adult human primary dermal fibroblasts are grown in DMEM high glucose (Microgem, UK) supplemented with 10% fetal bovine serum (Sigma-Aldrich), 1% glutamine, 1% antibiotics (Lonza) up to confluence. For surface marker staining, cell populations were washed with 1x PBS and stained with the PE-anti-IL-17RA antibody (6B7, BD Biosciences), or with the PE-anti-CD217 antibody (BioLegend, San Diego, CA) or with the PE-anti-IL-17RA antibody (R&D Systems) with consistent results. Staining with matched isotype control Ig was used as a control (BD Bioscience). Acquisition was performed using an Attune Nxt (Life-Technologies) cytofluorimeter. Analysis was performed using Flow logic software (Miltenyi, Germany).

1. Palombo, R.; Savini, I.; Avigliano, L.; Madonna, S.; Cavani, A.; Albanesi, C.; Mauriello, A.; Melino, G.; Terrinoni, A. Luteolin-7-glucoside inhibits IL-22/STAT3 pathway, reducing proliferation, acanthosis, and inflammation in keratinocytes and in mouse psoriatic model. *Cell Death Dis* **2016**, *7*, e2344.
